# Rupture of ectopic varices of the ascending colon occurring after pancreatic cancer surgery: A case report and literature review

**DOI:** 10.1002/deo2.255

**Published:** 2023-07-10

**Authors:** Yushi Inomata, Takeo Naito, Takashi Hiratsuka, Yusuke Shimoyama, Rintaro Moroi, Hisashi Shiga, Yoichi Kakuta, Kimiko Kayada, Yuki Ohara, Naoki Asano, Shuichi Aoki, Michiaki Unno, Atsushi Masamune

**Affiliations:** ^1^ Division of Gastroenterology Tohoku University Graduate School of Medicine Miyagi Japan; ^2^ Department of Surgery Tohoku University Graduate School of Medicine Miyagi Japan

**Keywords:** ascending colon varices, colon varices, ectopic varices, left‐sided portal hypertension, pancreatic cancer

## Abstract

A 69‐year‐old woman, a long‐term survivor of subtotal stomach‐preserving pancreatoduodenectomy with the splenic vein resection for pancreatic cancer, visited our hospital with a chief complaint of bloody stools. Previously, she was diagnosed with varices in the ascending colon due to left‐sided portal hypertension after pancreatoduodenectomy by computed tomography and colonoscopy. After emergency hospitalization, she went into shock, and blood tests showed acute progression of severe anemia. Computed tomography showed a mosaic‐like fluid accumulation from the ascending colon to the rectum. She was diagnosed with ruptured varices in the ascending colon. Emergency colonoscopy was performed, and treatment with endoscopic injection sclerotherapy using *N*‐butyl‐2‐cyanoacrylate was successful. Ectopic varices occur at any location other than the esophagus and stomach, and colonic varices are rare among them. They are mostly caused by portal hypertension due to liver cirrhosis. However, with the trend of improving the prognosis for patients with pancreatic cancer, we should occasionally pay attention to the development of ectopic varices including colonic varices in patients who have undergone pancreatoduodenectomy with superior mesenteric and splenic veins resection. Treatment methods for colonic varices varied from case to case, including conservative therapy, interventional radiology, and endoscopic procedure. In this case, endoscopic injection sclerotherapy was successfully performed without any complications. To the best of our knowledge, this is the first study to report successful treatment with endoscopic injection sclerotherapy for varices in the ascending colon caused by left‐sided portal hypertension after pancreatoduodenectomy. Colonic varices should be considered in patients with obscure gastrointestinal bleeding after pancreatoduodenectomy.

## INTRODUCTION

Ectopic varices occur at any location other than the esophagus and stomach.^1^ Watanabe et al. reported that in 173 cases of ectopic varices, the most common occurrence sites were the rectum (44.5%) and duodenum (32.9%), and only 6 (3.5%) cases occurred in the colon.^2^ Most cases of colonic varices are caused by portal hypertension due to liver cirrhosis; however, portal hypertension‐induced colonic varices due to surgical resection with vascular reconstruction for pancreatic cancer are even rarer. Given this rare occurrence, no standard treatments for colonic varices have been established. Therefore, various treatment methods could be considered appropriate for each case.

In this study, we report a case of excessive lower gastrointestinal bleeding that was caused by ruptured colonic varices due to portal hypertension after surgery for pancreatic head cancer and was treated successfully by endoscopic injection sclerotherapy (EIS) using *N*‐butyl‐2‐cyanoacrylate (NBCA).

## CASE PRESENTATION

**FIGURE 1:**
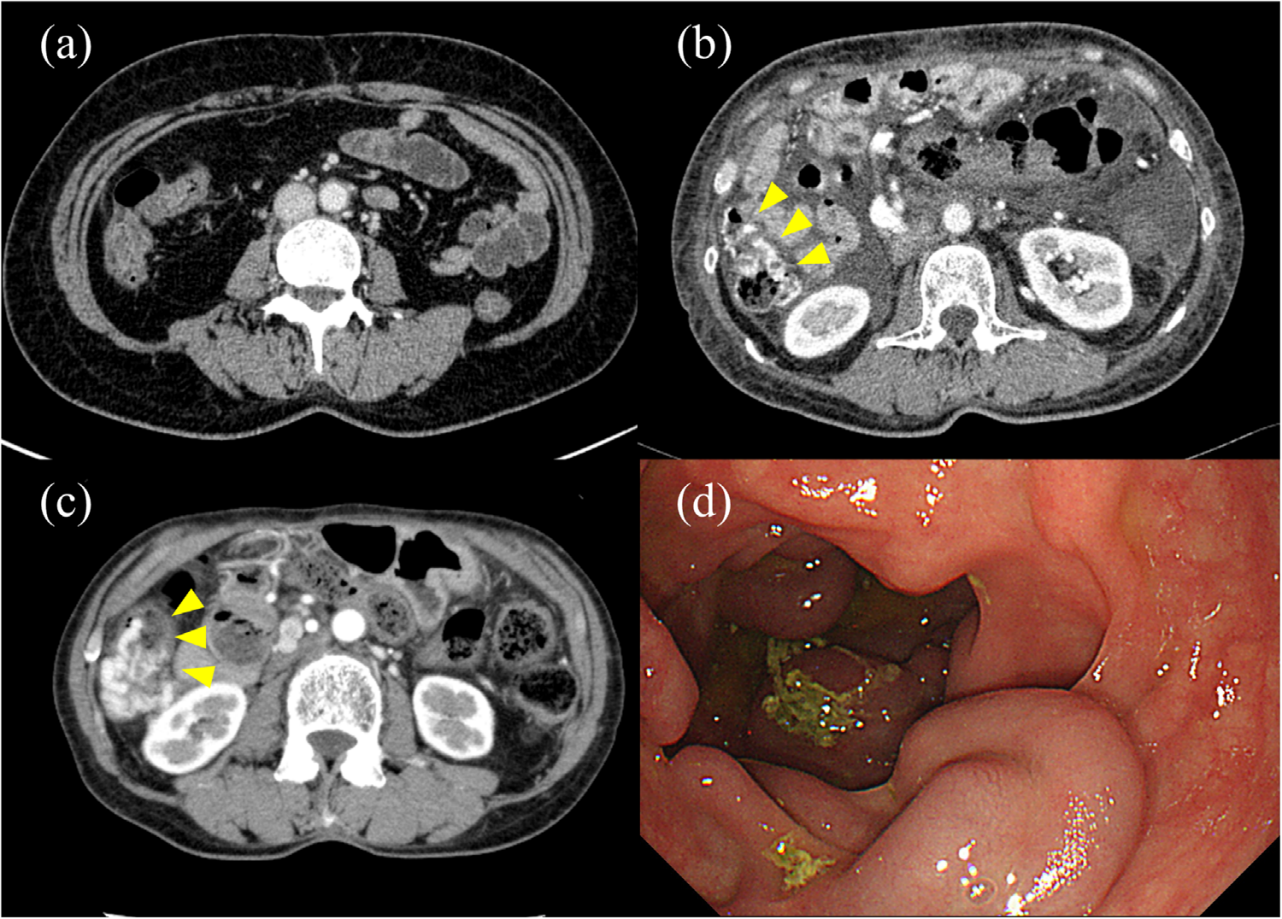
Imaging findings before variceal rupture. (a–c) Contrast‐enhanced computed tomography scan of the abdomen: (a) 6 months after surgery, (b) 1 year after surgery, and (c) 11 years after surgery (January 2022). With the development of collateral vessels, vasodilatation around the ascending colon and vascular structures on the mucosal surface were apparent (yellow arrowhead). (d) Colonoscopy revealed F3‐equivalent tortuous varices, but no red color signs.

In July 2022, a 69‐year‐old woman visited our hospital with a chief complaint of bloody stools. In December 2011, she underwent a subtotal stomach‐preserving pancreatoduodenectomy and reconstruction of the superior mesenteric vein with the splenic vein resection for pancreatic head cancer. The pathological diagnosis was pT3N0M0, which was equivalent to pStage IIA according to the 7th edition of the Union for International Cancer Control. After surgery, she received gemcitabine monotherapy as adjuvant chemotherapy; although a mass with suspected liver metastasis appeared, it gradually shrank with continued treatment. After the chemotherapy in December 2016, any recurrence had not been noticed. After surgery, contrast‐enhanced computed tomography (CE‐CT) revealed venous dilatation in the ascending colon, which gradually increased (Figure 1a–c). However, no evidence of cirrhosis was found. When colonoscopy was performed in March 2022 to check for chronic diarrhea, ectopic varices equivalent to F3 were found on the distal side of the ascending colon, but there were no bleeding and red color signs (Figure 1d).

**FIGURE 2:**
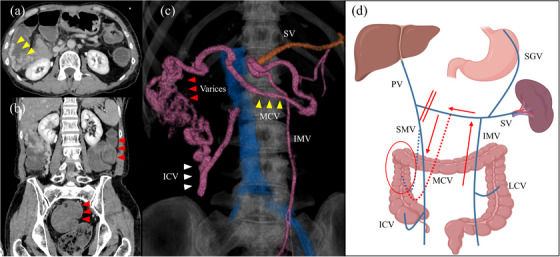
Abdominal contrast‐enhanced computed tomography at the time of variceal rupture. (a) Axial section: Although fluid collection and varices were present in the ascending colon, no leakage of contrast medium was seen at the varices (yellow arrowhead). (b) Coronal section: Large mosaic‐like fluid collection was seen on the left side of the colon, including the distal colon (red arrowhead). (c) 3D vascular image: The middle colonic vein as well as the inferior vein are connected to the resected splenic vein (yellow round circle). Dilated veins were seen in the route, the middle colonic vein (yellow arrowhead)→varices (red arrowhead)→the ileocecal vein (white arrowhead). (d) Schema of vascular anatomy (created with BioRender.com): For an anatomic anomaly of the middle colonic vein, originally should return to the superior mesenteric vein (blue dotted line), leading to the splenic vein (red dotted line), blood flow from the inferior mesenteric vein back to the splenic vein was flowing into the middle colonic vein and forming varices. PV, portal vein; SV, splenic vein; SMV, superior mesenteric vein; IMV, inferior mesenteric vein; LCV, left colic vein; MCV, middle colic vein; ICV, ileocolic vein; SGV, short gastric vein.

On the morning of the day before her visit, she had four bloody stools without abdominal pain, and the stools returned to normal within the day. However, it flared up again on the morning of the day of her visit. On admission, blood tests showed a slight decrease in hemoglobin from 11.2 to 9.9 g/dl compared with values 3 months earlier, but the blood urea nitrogen level was not elevated. Hepatic and renal functions were normal. Because several hours had passed since the last bloody stool, the patient was admitted for conservative treatment. However, she was immediately transported to a critical care center because of consciousness loss and shock vitals soon after admission. Blood tests showed a marked decrease in hemoglobin with 4.9 g/dl in a short time. CE‐CT showed a mosaic‐like fluid accumulation from the ascending colon to the rectum (Figure 2a,b). She had an anatomic anomaly of the middle colonic vein. It originally should return to the superior mesenteric vein and merge with the splenic vein. But a shunt was formed following splenic vein resection as shown in the figure, the feeding vein of varices was the middle colonic vein, and the draining vein was the ileocolic vein (Figure 2c,d).

**FIGURE 3:**
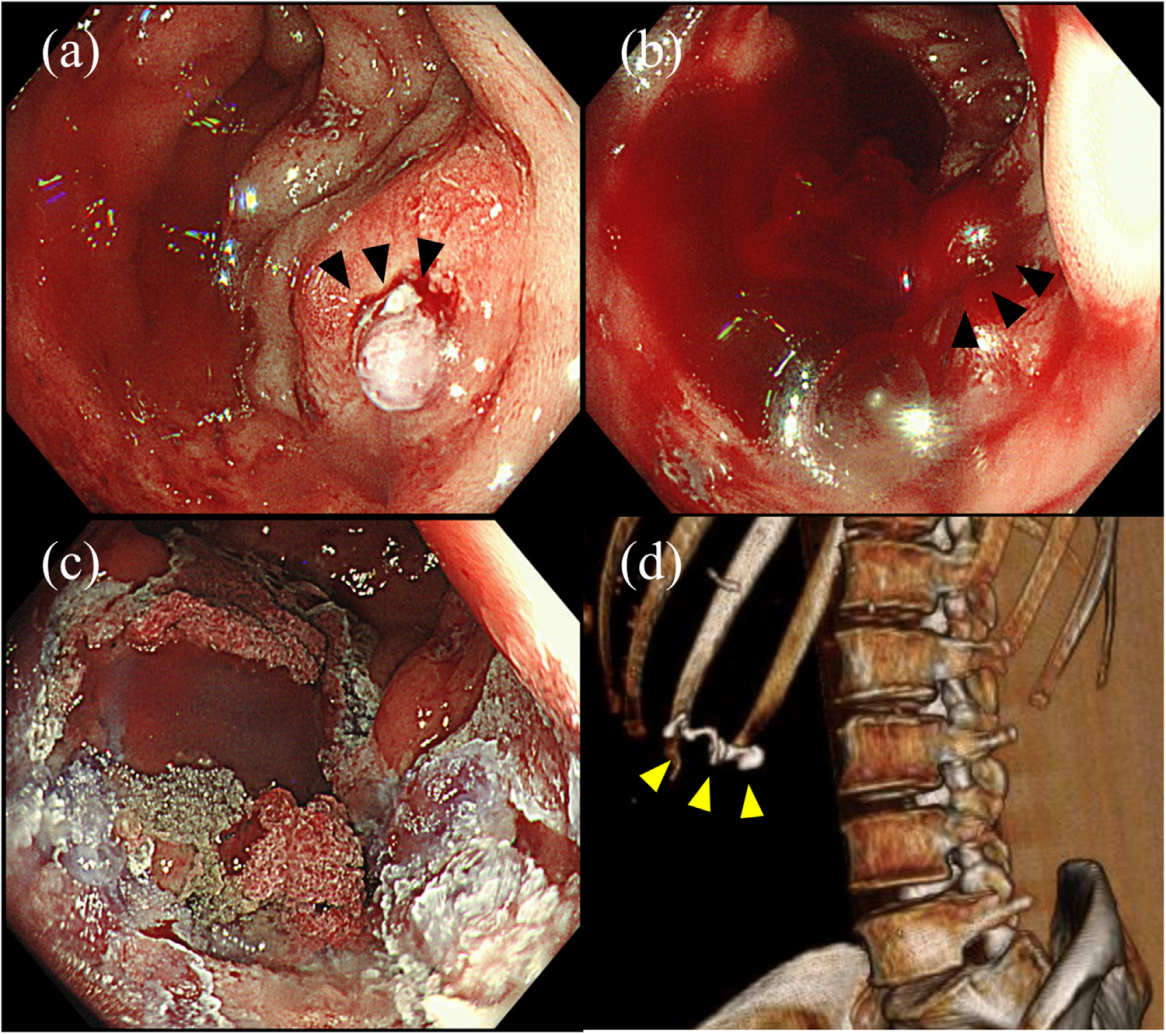
Imaging findings during endoscopic treatment. (a–c) Emergency colonoscopy. (a) Fibrin plug in the erosion was observed at the anorectal end of the varices (black arrowhead). (b) After puncturing the same site, injecting 2 ml of *N*‐butyl‐2‐cyanoacrylate (NBCA), and removing the needle, active bleeding occurred from the puncture site (black arrowhead). (c) After injecting another 1.6 ml of NBCA, the bleeding stopped. (d) Simple computed tomography (3D image) after treatment: the contrast medium was retained in the varix area, and no leakage into other vessels was observed.

Based on the above, we diagnosed the patient with hemorrhagic shock due to massive bleeding caused by the rupture of ectopic varices. After the patient was fully informed, an emergency colonoscopy was performed under general anesthesia for hemostasis. The normal lower endoscope (PCF‐H290ZI; Olympus Corporation, Tokyo, Japan) was used, without a tip cap. The emergency colonoscopy showed large amounts of fresh blood in the colon and a visible fibrin plug with blood oozing at the top of varix in the ascending colon, which was indicative of colonic varix with recent bleeding (Figure 3a). 75% NBCA was injected into varix with a 20‐g needle (Endoscopic Puncture Needle; TOP, Tokyo, Japan), resulting in the initial active spurting bleeding from the sites of the fibrin plug and injection (Figure 3b), which resolved after additional injection (Figure 3c). In total, 3.6 ml of 75% NBCA was injected, and contrast media retention was confirmed with a mobile X‐ray fluoroscopy system. Subsequent simple CT confirmed that the contrast media remained in the same site (Figure 3d). The patient had no recurrent bleeding and was discharged 9 days later. During her outpatient visit in September 2022, the hemoglobin level had improved to 10.9 g/dl, and the patient is currently under observation as of April 2023.

## DISCUSSION

In this case, a long‐term survivor after subtotal stomach‐preserving pancreatoduodenectomy for pancreatic cancer was successfully treated by EIS using NBCA for the emergent situation of a rupture of ectopic varices in the ascending colon. We searched PubMed using the keywords “Ascending colon and varices” and reviewed six cases of ruptured varices with detailed information since 2010 (Table 1).^3^, ^4^, ^5^, ^6^, ^7^, ^8^

**TABLE 1:** Literature review.

**Author**	**Year**	**Age**	**Sex**	**Background**	**Treatment**
Ishikawa et al.^3^	2021	57	M	PaC, after SSPPD+portal and splenic vein resection	Obliteration via a trans‐hepatic portal‐venous approach.
Murakami et al.^4^	2020	55	M	PaC, SMV obstruction due to tumor invasion	Transileocolic vein obliteration method.
Liu et al.^5^	2020	55	M	LC, HBV	Balloon occluded retrograde transvenous obliteration
Sousa et al.^6^	2016	50	M	LC, alcohol	EIS, using NBCA
Ko et al.^7^	2013	38	F	LC, alcohol	Venous embolization and EIS, using NBCA
Sohn et al.^8^	2012	33	F	SLE, thrombo‐occlusion of the left iliac vein	Conservative treatment (Octreotide and β‐blocker)

Abbreviations: EIS, endoscopic injection sclerotherapy; HBV, hepatitis B virus; LC, liver cirrhosis; NBCA, *N*‐butyl‐2‐cyanoacrylate; PaC, pancreatic cancer; SLE, systemic lupus erythematosus; SMV, superior mesenteric vein; SSPPD, subtotal stomach‐preserving pancreatoduodenectomy.

In the past, varices were often caused by underlying cirrhosis, but in recent years, some studies have reported pancreatic cancer‐related varices. When pancreatoduodenectomy for pancreatic cancer is performed, the splenic vein often needs to be resected to achieve tumor clearance and is rarely reconstructed. The division of the splenic vein can lead to left‐sided portal hypertension,^3^ which can cause ectopic varices including the colon. Mizuno et al. noted that the incidence of varices in the splenic vein resection group increased rapidly year by year up to 3 years postoperatively, reaching a plateau in about 40% of the patients.^9^ However, this report was based on CT rather than on endoscopic evaluation; thus, it is difficult to estimate the exact incidence rate of colonic varices, which are large enough to be detected by colonoscopy after pancreatoduodenectomy. Furthermore, they stated that this splenic vein resection is a bleeding risk for varices (including ectopic varices) that occur after pancreatoduodenectomy with portal vein resection. However, considering recent improvements in the prognosis for pancreatic cancers with the development of chemotherapy and surgical techniques,^10^ the incidence of colonic varices after pancreatoduodenectomy is expected to increase.

Treatment methods for colonic varices varied from case to case, including conservative therapy with beta‐blockers, interventional radiology (IVR), endoscopic variceal ligation, and EIS. However, emergency IVR is difficult to perform, except in very limited facilities, and endoscopic treatment appears to be more accessible than IVR. In this case, since the patient was in hemorrhagic shock, rapid and certain hemostasis was needed. IVR was difficult because there was no obscure extravasation in the colon. Therefore, we selected endoscopic therapies and performed EIS rather than endoscopic variceal ligation considering the lower rebleeding rate of EIS than endoscopic variceal ligation. Eventually, EIS was successfully performed without complications. To our knowledge, only one case report described varices in the ascending colon treated by the EIS method,^6^ and the present study is the first to report successful treatment of varices in the ascending colon caused by portal hypertension after pancreatoduodenectomy with EIS. Although EIS can be one of the appropriate treatment methods for colonic varices, treatment strategies should be established based on the further accumulation of similar cases in the future.

In summary, we describe a rare case of varix rupture in the ascending colon caused by portal hypertension after pancreatoduodenectomy. Therefore, colonic varices after pancreatoduodenectomy should be considered for patients with obscure gastrointestinal bleeding, and the EIS is an effective method for colonic varices.

## CONFLICT OF INTEREST STATEMENT

None.
